# Experiences of Long Ago: Erysipelas

**Published:** 1910-05-28

**Authors:** John Beddoe


### EXPERIENCES OF LONG AGO: ERYSIPELAS.

By JOHN BEDDOE, LL.D., M.D., F.R.C.P., F.R.S.

The things I am going to talk about happened in what I may call the pre-bacterial period, when medical practice was far more exclusively empirical than it is now. Probably the misanthropic organisms that have to do with erysipelas were greatly more abundant in my Edinburgh Infirmary and Dispensary days than they are now, whether by reason of the constant warfare waged against them by sanitarians, or whether partly from other causes, I do not know. But certainly erysipelas of the head and face, as well as erysipelas in connection with wounds, was common and formidable, and its treatment was a subject of much searching of brains.

So far as I am aware, it was Professor Hughes Bennett who first brought the tincture of muriate of iron, as it was then called, into vogue as a kind of specific for erysipelas. Since that time it has been quoted in most text-books, if not as a specific, yet as a standard or useful remedy; though some authors coolly inform us that cases of erysipelas and pneumonia are pretty sure to recover if you will only allow them to do so. The perchloride of iron, as it is now called, is lauded faintly by Watson Cheyne, in Allbutt and Rolleston's "System of Medicine"; he also mentions camphor, but not aconite, which, however, Marcus Beck, in Quain's "Dictionary," cites in its place as the second of only two available drugs. Most writers agree in recommending alcoholic stimulants.

My experiences with the perchloride were satisfactory for many years, but the time came when it failed in my hands, my patient succumbing apparently from extension to the meninges. During my residence as physician's assistant in the Edinburgh Infirmary, in the year 1854, I was one morning asked by my colleague, Dr. (now Sir John) Kirk, to see with him a severe case of erysipelas of the head, which he had admitted on the previous evening and about which he felt anxious. When we arrived in the ward he hesitated to identify his patient, whose face had been crimson and enormously swollen. The nurse, being appealed to, pointed out a *pale-faced* man, whose pulse and temperature were no more alarming than his complexion and general aspect. We looked at Dr. Kirk's prescription: it was a mild febrifuge (of nitre and mindererus, I think), flavoured with 10 minims of tincture of orange, and to be administered every second hour. "Did he take every dose, nurse?" "Yes, sir, he did, but he vomited it every time." We adjourned to the dispensary, where we made out that the prescription had gone through the hands of a raw apprentice, who had read *aconiti* for *aurantii*, and concocted the medicine accordingly. If the patient had not vomited it, I presume he would have been killed; as it was, he was cured, whether by the aconite or the vomiting I will not venture to say. I never had the courage to repeat the experiment.

Years afterwards Mr. Greenley, the oldest practitioner in Bristol, and a staunch teetotaler, who, partly perhaps because I too was an abstainer, usually consulted me when he found himself "in dubiis trepidisque rebus," came to ask my assistance in a case of erysipelas of the head. "He's an old gentleman of 78," he said, "a healthy man and rather abstemious; but he's going to die." We saw him together, and retired into an adjoining room. "Well, you too think he'll die, don't you?" "I do; but I wouldn't let him die without doing something more for him." "What would you like me to do?" "Alcohol is a good medicine, though a bad food; I would give him about as much brandy as you can get him to swallow." "Well, if you think so, it shall be done. Please meet me tomorrow morning at 10, if he's alive; I will let you know in time if he's dead, which I fear he will be." Hearing no tidings, I went next morning to King Square accordingly. My old friend met me with a smile. "Do you know, Doctor, I really believe he's going to get well." He did get well. How much brandy he had taken I forget, but it was a very large quantity, and I could not doubt that it saved his life.

